# A comparative study of an on premise AutoML solution for medical image classification

**DOI:** 10.1038/s41598-024-60429-4

**Published:** 2024-05-07

**Authors:** Kabilan Elangovan, Gilbert Lim, Daniel Ting

**Affiliations:** 1 Artificial Intelligence and Digital Health Research Group, Singapore Eye Research Institute, Singapore, Singapore; 2 Artificial Intelligence Office, Singapore Health Service, Singapore, Singapore; 3 Duke-NUS Medical School, National University of Singapore, Singapore, Singapore; 4 Singapore National Eye Centre, Singapore General Hospital, 11 Third Hospital Avenue, Singapore, 168751 Singapore; 5 Byers Eye Institute, Stanford University, Stanford, USA

**Keywords:** Medical imaging, Cancer imaging, Skin cancer, Cancer, Eye diseases, Neurological disorders, Respiratory tract diseases, Skin diseases

## Abstract

Automated machine learning (AutoML) allows for the simplified application of machine learning to real-world problems, by the implicit handling of necessary steps such as data pre-processing, feature engineering, model selection and hyperparameter optimization. This has encouraged its use in medical applications such as imaging. However, the impact of common parameter choices such as the number of trials allowed, and the resolution of the input images, has not been comprehensively explored in existing literature. We therefore benchmark AutoKeras (AK), an open-source AutoML framework, against several bespoke deep learning architectures, on five public medical datasets representing a wide range of imaging modalities. It was found that AK could outperform the bespoke models in general, although at the cost of increased training time. Moreover, our experiments suggest that a large number of trials and higher resolutions may not be necessary for optimal performance to be achieved.

## Introduction

Machine learning has made significant inroads into medicine in recent years^1^, with numerous successful applications in various sub disciplines such as radiology^2^, ophthalmology^3^ and pathology^4^ recognized by renowned medical journals. Such studies have tended to involve the classification of medical images, due to the relatively well-developed ability of deep learning models to automatically infer relevant features from sufficiently large quantities of annotated image data. In general, some deep learning model or ensemble of models is selected or designed by the study organizers, possibly after some preliminary experimentation, which is then trained to provide the diagnosis.

However, with the proliferation of interest in machine learning techniques within the wider medical community, there has been a rising demand for convenient and reliable automated machine learning (AutoML) frameworks that can be deployed for new tasks and data, without necessarily requiring machine learning experts to handle details such as pre-processing, feature selection, algorithm or model selection, and hyperparameter optimization. Ideally, such details and choices would be systematically tuned by an AutoML framework after the data and labels are defined, allowing an ML model to be generated with no particular programming or deep learning knowledge being required. Other than allowing the rapid prototyping of benchmark models, AutoML may also potentially reduce human bias in ML model choice and evaluation, by impartially exploring a large range of up-to-date model architectures and setups. Indeed, AutoML has obtained performances comparable to the state-of-the-art in some image classification tasks^5^.

Despite the potential of AutoML, however, there has been relatively little prior work examining its efficacy for medical imaging. This may be partly due to the synergy of AutoML with abundant computational resources due to its reliance on comprehensive searching through the model space, which has likely encouraged research on cloud platforms such as Google AI, Amazon Sagemaker, Microsoft Azure and H2O. However, the usage of such external platforms is often challenging for medical data in practice, due to patient privacy concerns. Still, much early investigation on AutoML in medical imaging has been performed on the Google platform^6–8^, with Faes et al. demonstrating comparable performance between AutoML and individual deep learning models, on five publicly-available open-source medical image datasets in 2019^9^. Korot et al. further compared six commercial AutoML platforms on four ophthalmic datasets, in 2021^10^, with the results suggesting that the choice of platform remains important.

Locally-executable general AutoML frameworks such as AutoKeras^11^, Auto-PyTorch^12^ and Auto-sklearn^13^ have since become more accessible, and have been benchmarked against both traditional machine learning models, and cloud platforms. On medical claim data, Romero et al. evaluated three AutoML tools (Auto-sklearn, H2O and TPOT) against a baseline random forest model, and concluded that the AutoML models performed similarly to each other, and better than the baseline^14^. Schwen et al. found that AutoGluon and Google AutoML Vision performed comparably to the best results known in the literature, for three histological image classification tasks^15^. Dael et al. demonstrated fusion on AutoML model subsets on stem cell, brain tumour and prostate cancer medical image datasets, using the Auto Tuned Models framework^16^. Yang et al. introduced MedMNIST^17^, a collection of ten pre-processed datasets of 28 × 28 medical images, and provided initial results from ResNet, Auto-sklearn, AutoKeras and Google AutoML Vision. AutoKeras has been applied to prostate cancer malignancy detection from multiparametric magnetic resonance images^18^, and also malaria detection from blood smear images^19^.

While a number of AutoML benchmarking studies for medical imaging have been performed as surveyed above, there may remain some gaps in the literature that we aim to fill in this study. Firstly, the focus of previous benchmarking efforts has often been focused on comparing multiple AutoML tools^10,15,20,21^, and less against a variety of commonly-used deep learning models. We therefore report performance against several popular deep learning architectures—VGG16, InceptionV3, DenseNet201 and ResNet50^22–25^—to provide more context about the added value of AutoML over typical use-cases. Secondly, the computational trade-off for AutoML has not often been considered, and is especially relevant for local implementations with relatively limited resources. We therefore examine the impact of varying the number of trials allowed during training. Thirdly, the resolution of medical images is known to affect diagnosis accuracy, but the computation costs of AutoML compared to single-model deep learning has encouraged the use of lower resolutions and image sizes for AutoML. We therefore also consider the effect of different image resolutions, on AutoML performance.

## Methods

### AutoKeras overview

AutoKeras (AK), an open-source AutoML library developed by the DATA Lab at Texas A&M University^11^, offers a powerful and user-friendly approach to automated machine learning. It utilizes Bayesian Optimization^26,27^, a mathematical technique, to optimize model parameters without the need for derivative calculations. By generating queries and learning from the performance, AK finds the best model architecture.

The Bayesian method consists of three stages: generating architectures, training them, and updating the learned probability distribution. To improve training times, AK employs graph-level morphism, which morphs parent networks into child networks, and layer-level morphism, which systematically explores and morphs layers affected by single-layer mutations.

AK pre-processes datasets by normalizing and augmenting them before passing them to the model searcher. The model search algorithm runs on the CPU, while model training occurs in parallel on the GPU. AK adapts to varying memory sizes and trains models that fit within the memory limit. The AK image classifier starts with a simple model and iteratively mutates it. Once convergence or the time limit is reached, the best model is saved, and a final training phase fine-tunes the model using the validation set. The resulting AK model can be exported as a Keras model for visualization or inference purposes.

### Data source

**Figure 1:**
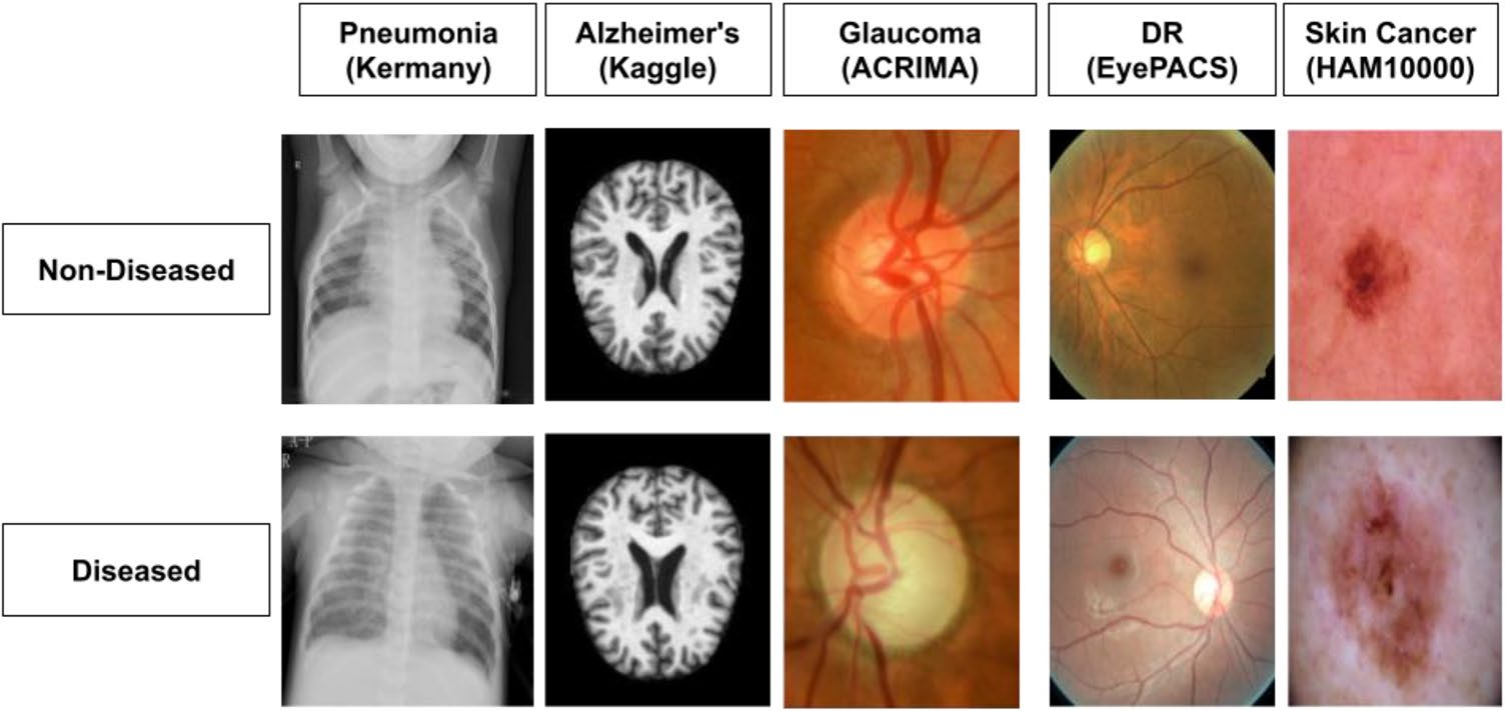
Sample images for disease classification (diseased vs non-diseased).

We utilized five distinct open-source datasets from Kaggle, comprising medical images to develop deep learning models for disease diagnosis across various medical specialties (Fig. 1). This approach ensured a robust evaluation of diagnostic accuracy for a diverse range of conditions. The datasets include:

1. Chest X-Ray (CXR) images for Pneumonia diagnosis^28^: Facilitating the binary classification of diseased and non-diseased classes for pneumonia detection.
2. Brain Magnetic Resonance Imaging (MRI) images for Alzheimer's diagnosis^29^: Aids in Alzheimer's disease detection through binary classification, with certain adaptations for combining relevant classes.
3. Retinal fundus images from EyePACS for Diabetic Retinopathy (DR) diagnosis^30^: These images are part of the EyePACS dataset and are structured for binary classification in detecting DR.
4. Retinal fundus images from ACRIMA for Glaucoma diagnosis^31^: Provides images for binary classification tasks in glaucoma detection.
5. Skin Mole images from HAM10000 for Cancer Detection^32^: Used for the binary classification of skin cancer.

These datasets were meticulously structured for binary classification tasks, with specific adaptations made for DR and Alzheimer’s datasets to combine relevant classes. The breakdown of each dataset into training, validation, and test sets is detailed in Table 1 of our study, providing a clear understanding of the experimental data setup.

**Table 1:** Datasets summary and breakdown for deep learning modelling.

Dataset	Medical specialty	Task	Train	Validation	Test
Chest X-ray (Kermany)	Pulmonology	Healthy vs pneumonia	Healthy—1260 Pneumonia—1047	Healthy—180 Pneumonia—149	Healthy—360 Pneumonia- 360
Brain MRI (Kaggle)	Neurology	Non-Dem vs Dem	Non-Dem—2304 Dem—2304	Non-Dem—256 Dem—257	Non-Dem—640 Dem—639
Retinal fundus (EyePACS)	Ophthalmology	Non-RefDR vs RefDR	NonRefDR—7839 RefDR—3826	NonrefDR—1120 RefDR—546	NonRefDR—2241 RefDR—1094
Retinal fundus (ACRIMA)	Ophthalmology	Healthy vs glaucoma	Healthy—216 Glaucoma—277	Healthy—30 Glaucoma—39	Healthy—63 Glaucoma—80
Skin mole (HAM10000)	Dermatology	Benign vs malignant	Benign—1260 Malignant—1047	Benign—180 Malignant—149	Benign—360 Malignant—301

### Experimental platform

In this study, the Windows 10 Pro system platform with 64 bits was used as the software platform for implementing the experiments of this research, where the hardware consisted of dual GPU system with 24 GB VRAM with NVIDIA RTX 3090, and the Intel(R) also was used as a hardware component with Intel(R) Core(TM) i9-10920X CPU @ 3.50 GHz. The experiments were performed in the environment of a Jupyter Notebook with Python programming language Version 3.8.13.

### Experimental design

In this study, we conducted 3 experiments in total for comparative analysis with standardised experimental constraints.

#### Experiment 1: AK vs bespoke CNN models (single GPU)

In this experiment, our objective was to validate and assess the accuracy of AK in effectively classifying five different classes of diseases. We aimed to compare its performance with CNN models, namely VGG16, InceptionV3, DenseNet201 and ResNet50. These CNN models were specifically chosen because they are not commonly found in model zoos or image block specifications with their documentation. To train the models, we utilized a single RTX 3090 GPU Card and kept the parameters consistent.

For the AK training configuration, we employed the ImageClassifier class to compile and build the image classification model for all five datasets. We used default parameters for this purpose. In our case, the ImageClassifier accepted inputs of size 224 × 224 × 3. It iteratively selected from a range of building blocks, including ResNet^25^, ResNext^33^, Xception^34^, EfficientNets^35^, and simple CNNs. This allowed the construction of neural networks with varying complexity, depth, and data augmentation pipelines. ImageClassifier was then fitted to the training and validation datasets with evaluation metrics set as “binary_accuracy”, with max_trials to 10 and trained the models for 50 epochs. The max_trials parameter determines the number of architectures AK trains before determining the best model.

For the Bespoke CNN models, we utilized the concept of transfer learning, a widely used approach in deep learning. Transfer learning involves leveraging a pretrained model as the starting point for a new model on a different task^36^. In our case, we employed three popular transfer learning CNN models: VGG16, InceptionV3, DenseNet201 and ResNet50, all pretrained on the ImageNet dataset^37^. To implement these models, we used the Keras API, configuring them as base models with specific parameters. The parameters included setting the weights as “imagenet”, excluding the top layer (include_top = False), and defining the input shape as (224, 224, 3). This established the foundation of the models for our experiment. The output from the base models was then connected to a global average pooling 2D layer, which summarizes the spatial information, and further linked to a final dense layer. The activation function for the dense layer was set as “sigmoid” for binary classification. We compiled the models using a batch size of 32, the “Adam” optimizer with a learning rate of 1 × 10^−3^, and evaluated the performance using the 'binary_accuracy' metric. The training process consisted of 50 epochs, with early stopping implemented to minimize validation loss. Specifically, if no improvement was observed for 10 consecutive epochs, the training would stop.

#### Experiment 2: AK_10 vs AK_100 (Dual GPU)

In this experiment, we conducted training for two sets of AK models. The first set had a maximum trial count of 10, while the second set had a maximum trial count of 100. We trained these models on all five datasets, using the same training configuration as described in Experiment 1. Notably, for this experiment, we utilized two GPU cards to expedite the training process and improve efficiency. By exploring different trial counts, we aimed to observe the impact of the maximum trial count on the performance and accuracy of the AutoKeras models across the various datasets.

#### Experiment 3: AK_10 resolution experiments (dual GPU)

In this experiment, we focused on training models using three resolution settings: (224 × 224), (150 × 150) and (75 × 75), for each of the five datasets. The training process utilized two GPU cards to enhance efficiency. However, we limited the max_trials to only 10 with AK. The objective was to observe any noteworthy findings or insights that could arise from these resolution experiments. By exploring the impact of resolution on model performance, we aimed to gain a better understanding of the relationship between image resolution and classification accuracy across diverse datasets.

### Evaluation metrics

The performance of the image classification models on the test dataset using binary_accuracy @0.5 threshold and AUC metrics, along with the F1, Precision, and Recall scores (including their macro averages) calculated at a 0.5 threshold. Our study emphasizes the models’ discriminatory power by reporting threshold values and specificity, while ensuring a fixed sensitivity of 90%. This method allows for an extensive assessment of the classification models, covering both AutoKeras (AK) and Bespoke Models. This method enables a more thorough evaluation of the classification models, encompassing both AutoKeras (AK) and Bespoke Models, ensuring a broad and definitive assessment.

By focusing on the ability to maintain high sensitivity while achieving a specific level of specificity, we address the clinical implications of the classification models. In medical diagnostics, it is crucial to strike a balance between accurately identifying positive cases (high sensitivity) and minimizing false positives (high specificity)^38^. A fixed sensitivity of 90% ensures that the models can consistently capture a significant portion of positive cases, while evaluating the specificity allows us to assess their performance in accurately ruling out negative cases. In order to determine the threshold that optimizes specificity while achieving a desired sensitivity level. Our method involves calculating the confusion matrix and sensitivity initially at 0.5 threshold value. If the calculated sensitivity falls below the desired value of 90, the threshold is adjusted using the receiver operating characteristic (ROC) curve otherwise the specificity will be calculated at 0.5 threshold. The updated threshold is then used to obtain binary predictions, and a new confusion matrix is computed to evaluate the model’s specificity. We have a compelling reason for taking this approach: in binary classification, the conventional threshold value is typically set at 0.5. Therefore, if the model is already capable of achieving a sensitivity of 90% or higher at the 0.5 threshold, we will accurately report the corresponding calculated specificity.

This comprehensive evaluation has important clinical implications. Models with high sensitivity and specificity at a fixed threshold can aid in improving diagnostic accuracy, enabling early detection of diseases or abnormalities. The ability to differentiate effectively can reduce the chances of false positives, preventing unnecessary interventions or treatments for patients who are actually negative. This evaluation approach provides valuable insights into the clinical utility and practicality of both AK and Bespoke Models, assisting medical practitioners in making informed decisions regarding their implementation in healthcare settings.

## Results and evaluation

### Experiment 1

Based on the results of Experiment 1 (Table 2), both transfer learning with pretrained CNNs and AK demonstrated strong performance across the classification tasks. The AK models exhibited accuracies ranging from 0.62 to 0.98 and AUC scores ranging from 0.68 to 1.00. Notably, the AK models outperformed the bespoke CNN models in terms of accuracies, AUC, and specificity metrics in four out of five classification tasks, except for Alzheimer's classification, where DenseNet201 achieved an AUC of 0.77 and accuracy of 0.65 (Table 2).

**Table 2:** Experiment 1 results-1 (AutoKeras (AK) vs bespoke CNN models).

Model	Training time (mins)	AUC	ACC	F1	Precision	Recall
Pneumonia classification task						
AK	544	**1.00**	**0.98**	**0.97**	**0.96**	**0.98**
DenseNet201	27	0.98	0.94	0.93	0.92	0.93
InceptionV3	27	0.96	0.92	0.89	0.90	0.89
VGG16	51	0.96	0.84	0.83	0.81	0.88
ResNet50	**26**	0.90	0.80	0.70	0.78	0.68
Alzheimer’s classification task						
AK	206	0.68	0.62	0.62	0.62	0.62
DenseNet201	40	**0.77**	**0.65**	**0.63**	**0.71**	**0.65**
InceptionV3	36	0.70	0.63	**0.63**	0.64	0.63
VGG16	36	0.76	0.62	0.60	0.65	0.62
ResNet50	**12**	0.60	0.50	0.41	0.50	0.50
DR classification task						
AK	2901	**0.87**	**0.83**	**0.80**	**0.82**	**0.79**
DenseNet201	103	0.76	0.74	0.67	0.72	0.66
InceptionV3	104	0.74	0.73	0.64	0.72	0.63
VGG16	233	0.66	0.69	0.52	0.66	0.55
ResNet50	**44**	0.55	0.67	0.40	0.34	0.50
Glaucoma classification task						
AK	29	**1.00**	**0.98**	**0.98**	**0.98**	**0.98**
DenseNet201	5	0.98	0.94	0.94	0.95	0.94
InceptionV3	**3**	0.94	0.87	0.87	0.88	0.87
VGG16	4	0.93	0.80	0.79	0.83	0.78
ResNet50	5	0.83	0.56	0.36	0.28	0.50
Cancer classification task						
AK	91	**0.95**	**0.87**	**0.87**	**0.87**	**0.87**
DenseNet201	14	0.95	0.86	0.86	0.87	0.86
InceptionV3	**10**	0.93	0.86	0.86	0.86	0.85
VGG16	20	0.92	0.84	0.84	0.84	0.84
ResNet50	20	0.83	0.67	0.63	0.71	0.64

Bolded values indicate most favorable metrics.

However, it is important to consider that the training time for the AK models was longer compared to the bespoke models, with the longest time reported at 2902 mins for the DR classification task (Table 2). This can be attributed to the fact that AK models were trained from scratch without any layer freezing or utilization of pre-trained weights. It is worth mentioning that the threshold values remained relatively stable across all models, except for the AK models in the DR and Alzheimer's classification tasks. In these cases, the thresholds were lower, at 8.49E−5 and 2.00E−9, respectively (Table 3). The lower thresholds in AK models could potentially be attributed to the inconsistency in the datasets, which may have affected the model’s ability to distinguish between classes. In contrast, the bespoke CNN models, with their utilization of pre-trained weights and transfer learning techniques, performed better in these cases. Interestingly, while ResNet50 underperformed in all classification tasks, it yielded the highest threshold value, nearing the optimal 0.5, yet it also recorded one of the lowest specificity values. This indicates that ResNet50’s predictions were somewhat biased or one-sided (Table 3).

**Table 3:** Experiment 1 results-2 (autokeras (ak) vs bespoke CNN models).

Model	Specificity	Threshold
Pneumonia classification task		
AK	**0.98**	0.50
DenseNet201	0.91	0.50
InceptionV3	0.82	0.50
VGG16	0.90	0.37
ResNet50	0.71	**0.58**
Alzheimer’s classification task		
AK	0.31	2.00e-09
DenseNet201	0.48	0.14
InceptionV3	0.31	0.28
VGG16	**0.50**	0.29
ResNet50	0.11	**0.50**
DR classification task		
AK	**0.56**	8.49e−05
DenseNet201	0.34	0.16
InceptionV3	0.32	0.18
VGG16	0.22	0.19
ResNet50	0.12	**0.31**
Glaucoma classification task		
AK	**0.98**	0.50
DenseNet201	0.90	0.50
InceptionV3	0.81	0.50
VGG16	0.62	0.50
ResNet50	0.62	**0.52**
Cancer classification task		
AK	**0.84**	0.34
DenseNet201	0.83	0.30
InceptionV3	0.80	0.30
VGG16	0.77	0.34
ResNet50	0.65	**0.38**

Bolded values indicate most favorable metrics.

While AK demonstrated strong performance across multiple classification tasks, it is important to consider the trade-offs in terms of training time and dataset consistency. The bespoke CNN models, leveraging transfer learning, showed promising results, particularly in cases where dataset inconsistencies could potentially impact model performance.

### Experiment 2

In this experiment utilizing dual GPU training, it is evident that the training time of the AK_10 model has been significantly reduced compared to the results obtained in Experiment 1 (Table 4). Interestingly, minimal to no improvements in evaluation metrics (AUC, accuracy, specificity) were observed between the AK_10 (10 Trials) and AK_100 (100 Trials) models, and these differences were not statistically significant. Another notable observation is that, after conducting either 10 or 100 trials, the selected "best" model architectures primarily consisted of EfficientNetB7 for the Pneumonia and DR classification tasks, while custom Vanilla CNNs were preferred for the other tasks. The observation is intriguing considering that Vanilla CNNs are generally characterized by less complex architectures and a lower number of trainable parameters. It is noteworthy that a Vanilla CNN with a parameter count of only 19,457 exhibited superior performance in terms of AUC and accuracy for the skin cancer classification task compared to other models in the experiment.

**Table 4:** Experiment 2 results-1 (AK_10 vs AK_100).

MODEL	Training time (mins)	AUC	ACC	F1	Precision	Recall	Model architecture	Parameters
Pneumonia classification task								
AK_10	**415**	**0.99**	**0.98**	**0.97**	**0.97**	0.97	EfficientNetB7	63, 789, 521
AK_100	5181	0.99	0.97	0.96	0.96	0.97	EfficientNetB7	63, 789, 521
Alzheimer’s classification task								
AK_10	**165**	0.73	0.66	**0.65**	0.67	0.66	Vanilla CNN	793, 793
AK_100	1085	**0.75**	0.66	0.64	**0.71**	0.66	Vanilla CNN	**397, 345**
DR classification task								
AK_10	**900**	**0.85**	0.81	0.77	0.78	**0.77**	EfficientNetB7	63, 789, 521
AK_100	2869	0.84	**0.80**	0.77	**0.79**	0.75	EfficientNetB7	63, 789, 521
Glaucoma classification task								
AK_10	**23**	**1.00**	**0.98**	**0.98**	**0.98**	**0.98**	Vanilla CNN	793,793
AK_100	115	0.98	0.97	0.96	0.97	0.96	Vanilla CNN	3, 252, 289
Cancer classification task								
AK_10	**49**	**0.96**	**0.87**	**0.86**	**0.87**	**0.86**	Vanilla CNN	19, 457
AK_100	3443	0.94	0.86	0.86	0.86	0.85	Vanilla CNN	2, 374, 657

Bolded values indicate most favorable metrics.

Additionally, with dual GPU training, the reported threshold values were more stable compared to those obtained in Experiment 1 using AK (Table 5). This stability indicates a reduced variability in the model's decision-making process. However, it is important to consider the trade-off between conducting 10 or 100 trials. The results suggest that comparable performance can already be achieved with just 10 trials using AK. Therefore, this approach proves to be more resource-efficient and offers a practical alternative for obtaining satisfactory results without the need for extensive trial runs.

**Table 5:** Experiment 2 results-2 (AK_10 vs AK_100).

Model	Specificity	Threshold
Pneumonia classification task		
AK_10	0.94	0.5
AK_100	**0.97**	0.5
Alzheimer’s classification task		
AK_10	0.38	0.018
AK_100	**0.42**	**0.5**
DR classification task		
AK_10	**0.52**	**0.15**
AK_100	0.48	0.12
Glaucoma classification task		
AK_10	**0.97**	0.5
AK_100	0.94	0.5
Cancer classification task		
AK_10	**0.87**	**0.35**
AK_100	0.86	0.31

Bolded values indicate most favorable metrics.

### Experiment 3

In this experiment, the variation in training image resolutions or input resolutions had a significant impact on the selection of the final “best” model across the trials conducted in AK, as well as the training time required for these trials (Table 6). The reason behind this impact lies in the allocation of GPU memory by AK when smaller input resolutions are specified.

**Table 6:** Experiment 3 results-1 (AK_224 vs AK_150 vs AK_75).

Model	Training time (mins)	AUC	ACC	F1	Precision	Recall	Model architecture	Parameters
Pneumonia classification task								
AK_224	**415**	**0.99**	**0.98**	**0.97**	**0.97**	**0.97**	EfficientNetB7	63, 789, 521
AK_150	520	0.99	0.97	0.96	0.96	0.95	EfficientNetB7	63, 789, 521
AK_75	750	0.99	0.97	0.96	0.96	0.96	EfficientNetB7	63, 789, 521
Alzheimer’s classification task								
AK_224	**165**	0.73	0.66	0.65	0.67	0.66	Vanilla CNN	793, 793
AK_150	199	0.76	**0.70**	**0.70**	0.70	**0.70**	Vanilla CNN	360, 449
AK_75	330	**0.78**	**0.70**	**0.70**	**0.72**	**0.70**	Vanilla CNN	97, 793
DR classification task								
AK_224	**900**	**0.85**	0.81	0.77	0.78	**0.77**	EfficientNetB7	63, 789, 521
AK_150	1140	**0.86**	**0.82**	**0.78**	**0.82**	0.77	EfficientNetB7	63, 789, 521
AK_75	990	0.79	0.76	0.70	0.74	0.69	EfficientNetB7	63, 789, 521
Glaucoma classification task								
AK_224	**23**	1.00	0.98	0.98	0.98	0.98	Vanilla CNN	793,793
AK_150	**12**	1.00	0.98	0.98	0.98	0.98	Vanilla CNN	360, 449
AK_75	18	1.00	0.98	0.98	0.98	0.98	Vanilla CNN	97, 793
Cancer classification task								
AK_224	**49**	**0.96**	0.87	0.86	0.87	0.86	Vanilla CNN	19, 457
AK_150	247	0.95	**0.88**	**0.88**	**0.88**	**0.88**	EfficientNetB7	63, 789, 521
AK_75	350	**0.96**	**0.88**	**0.88**	0.87	0.88	EfficientNetB7	63, 789, 521

Bolded values indicate most favorable metrics.

When a smaller input resolution is used, AK can allocate more GPU memory to the trials involving larger architectures such as EfficientNetB7. Consequently, in most cases, decreasing the resolutions from 224 to 150 to 75 resulted in an increase in training time for the trials (Table 6).

Notably, the threshold values for pneumonia, glaucoma, and cancer classification tasks consistently approached or matched the optimal value of 0.5 (Table 7). Furthermore, for Alzheimer’s classification at a lower resolution of 75 × 75, there was an observed increase in specificity and an associated threshold, improving to 0.52 and 0.24 respectively, compared to higher resolutions. This suggests that lower resolution training and inference can, in some instances, enhance results.

**Table 7:** Experiment 3 results-2 (AK_224 vs AK_150 vs AK_75).

Model	Specificity	Threshold
Pneumonia classification task		
AK_224	0.94	0.5
AK_150	0.93	0.5
AK_75	**0.95**	0.5
Alzheimer’s classification task		
AK_224	0.38	0.018
AK_150	0.43	5.36e−03
AK_75	**0.52**	**0.24**
DR classification task		
AK_224	0.52	**0.15**
AK_150	**0.53**	0.092
AK_75	0.40	0.13
Glaucoma classification task		
AK_224	0.97	0.5
AK_150	**0.98**	0.5
AK_75	**0.98**	0.5
Cancer classification task		
AK_224	**0.87**	0.35
AK_150	0.86	**0.39**
AK_75	0.85	0.25

Bolded values indicate most favorable metrics.

The results highlight that both EfficientNetB7 and Vanilla CNNs emerged as the best-selected models across the trials conducted in the resolution experiments. This observation underscores the effectiveness and suitability of these architectures for handling variations in input resolutions and their ability to adapt to different resolution settings.

## Discussion

The experimental results provide evidence to support the notion that a simple CNN architecture can achieve the “best” results without the need for complex CNN architectures. Several scientific factors can help explain this observation. Simple CNN architectures are often characterized by fewer layers and parameters compared to complex architectures, striking a balance between model complexity and generalization ability. While complex architectures may possess a higher capacity to capture intricate patterns, they can also be prone to overfitting, especially with limited data. In contrast, simple architectures can exhibit better generalization and avoid overfitting in scenarios where the dataset is relatively small or lacks diversity (e.g. Skin Cancer, Glaucoma datasets).

The principle of Occam’s Razor^39^ supports the idea of favouring simpler models when multiple explanations or models can account for a phenomenon. In the context of CNN architectures, Occam's Razor suggests that simpler models can be sufficient for achieving the “best” results, especially when considering factors such as dataset size and complexity. By adhering to this principle, researchers and scientists can prioritize simpler models that provide comparable or even superior performance to more complex counterparts. Additionally, simpler models offer an advantage during inference, as they require fewer computational resources and can make predictions in a shorter time.

However, it is important to note that the suitability of a CNN architecture depends on the specific task, dataset, and available resources. Complex architectures may be necessary to handle intricate patterns or large-scale datasets in certain scenarios. Nevertheless, the experimental results presented here provide evidence that, in certain cases, simplicity can be an advantageous attribute in CNN architectures. The development of AutoML has raised the possibility for medical practitioners to optimize classification models on their own, offering a promising solution. With the availability of open-source, locally-deployable AutoML frameworks such as AK, optimizing classification models becomes a streamlined process that can be executed with a single line of code. This simplicity eliminates the need for extensive programming knowledge and technical expertise, lowering the entry barrier for practitioners and researchers interested in leveraging machine learning for their applications. Furthermore, the utilization of these AutoML frameworks mitigates concerns regarding data privacy regulations. By keeping the model optimization process on-premise, sensitive patient data can remain secure within the healthcare environment. This alleviates potential compliance issues and ensures that the development and deployment of machine learning models adhere to strict privacy protocols.

Also, it is worth mentioning that despite the convenience and accessibility provided by AutoML frameworks like AK, the current literature lacks comprehensive analysis on benchmarking these frameworks, particularly with respect to common parameters of practical interest. In past literature, there has been a lack of extensive experimentation with AK to understand the impact of the number of trials and training image resolutions on training time and model performances.

To address this gap, we not only examine the effect of parameters such as the number of required trials and image input resolution on a wide range of medical image modalities in this study, but also investigated the resource efficiency in terms of computing resources and training time required when building models with AK. Limitations of the study include the limited granularity for the parameters being examined, and the possibility for interactions between the values of the different parameters, which were not tested. Furthermore, we plan to investigate the application of broader and more varied datasets in our subsequent research endeavours. The choice to utilize smaller datasets in the current study was strategic, aimed at reflecting scenarios where access to extensive data might not be readily available. This decision enabled us to specifically evaluate AutoKeras and other neural networks’ performance under conditions that are often a reality for researchers, where limited data is the norm. Our approach sought to highlight the efficiency and potential of AutoML technologies in less-than-ideal data situations, contributing valuable insights into their capabilities. Moving forward, we aim to expand our investigations to include more varied datasets, further enriching our understanding of these tools' performance across different data conditions. Nevertheless, we believe that the findings comprise a useful resource for researchers wishing to incorporate AutoML into their development pipelines.

## Conclusion

The computational cost is a crucial consideration when deploying deep learning models in healthcare. Our experiments using AK demonstrated that complex CNN architectures like EfficientNetB7 achieve high accuracy but require extensive trials, training time and substantial computational resources. However, our study showed that with just 10 trials using AK, we achieved state-of-the-art performance, often selecting custom Vanilla CNN architectures as the best models. This means that AK enables the development of on-premise DL models with the right computational resources, eliminating data privacy and cloud computing cost issues from commercial AutoML solution providers. These lightweight and simple CNN models can be easily built and deployed within healthcare, offering high diagnostic accuracy.

Understanding the trade-off between model performance and resource consumption is essential for informed decision-making in healthcare DL solutions. Our analysis provides comprehensive insights into the resource efficiency of AutoML frameworks like AK, guiding practitioners in selecting the most suitable approach for medical image classification. By optimizing classification models effectively and overcoming limited computational resources, our study aims to facilitate the adoption of resource-efficient DL models in healthcare. This advancement paves the way for more accessible and practical machine learning solutions, ultimately enhancing patient care and diagnostic accuracy.

## Data Availability

The datasets we used and analyzed during our study are open source and available from Kaggle, ordered according to your request: (1) Pneumonia Chest X-Ray data was retrieved from: https://www.kaggle.com/datasets/paultimothymooney/chest-xray-pneumonia. (2) Alzheimer’s Disease data was retrieved from: https://www.kaggle.com/datasets/tourist55/alzheimers-dataset-4-class-of-images. (3) Diabetic Retinopathy—EyePACS data was retrieved from: https://www.kaggle.com/competitions/diabetic-retinopathy-detection/data. (4) Glaucoma—ACRIMA data was retrieved from: https://www.kaggle.com/datasets/sshikamaru/glaucoma-detection. (5) Skin Cancer—HAM10000 data was retrieved from: https://www.kaggle.com/datasets/fanconic/skin-cancer-malignant-vs-benign.
